# Beware of vested interests: Epistemic vigilance improves reasoning about scientific evidence (for some people)

**DOI:** 10.1371/journal.pone.0231387

**Published:** 2020-04-15

**Authors:** Lukas Gierth, Rainer Bromme

**Affiliations:** Institute of Psychology, University of Münster, Münster, North Rhine-Westphalia, Germany; Universidad de Granada, SPAIN

## Abstract

In public disputes, stakeholders sometimes misrepresent statistics or other types of scientific evidence to support their claims. One of the reasons this is problematic is that citizens often do not have the motivation nor the cognitive skills to accurately judge the meaning of statistics and thus run the risk of being misinformed. This study reports an experiment investigating the conditions under which people become vigilant towards a source’s claim and thus reason more carefully about the supporting evidence. For this, participants were presented with a claim by a vested-interest or a neutral source and with statistical evidence which was cited by the source as being in support of the claim. However, this statistical evidence actually contradicted the source’s claim but was presented as a contingency table, which are typically difficult for people to interpret correctly. When the source was a lobbyist arguing for his company’s product people were better at interpreting the evidence compared to when the same source argued against the product. This was not the case for a different vested-interests source nor for the neutral source. Further, while all sources were rated as less trustworthy when participants realized that the source had misrepresented the evidence, only for the lobbyist source was this seen as a deliberate attempt at deception. Implications for research on epistemic trust, source credibility effects and science communication are discussed.

## Introduction

In a political talk show on May 1^st^, 2019, a German politician supported his party’s rejection of the mitigation of carbon emissions by citing the statistic that two thirds of climate studies are unsure about the causes of global warming. The use of this statistic is misleading however; only about one third of climate studies investigated the causes of global warming in the first place. Of these, 97.1% agreed that human-caused carbon emissions were the cause of global warming [1, 2]. This small incident illustrates one of the problems associated with the use of statistical evidence as a persuasive strategy: it is difficult for people to identify whether a piece of evidence actually supports the associated claim. Generally, people have difficulties in correctly interpreting statistics that are presented in a certain way [3]. This would be particularly problematic when statistical evidence is misrepresented on purpose by a source with a political or financial agenda.

For instance, the data contained in contingency tables such as Table 1 are difficult to interpret for people. The table shows that two thirds of people who took drug A get better, while of those who took drug B, three fourths get better. While such evidence should be damning for the pharma company who developed drug A, this would only be the case if people analyze the table correctly. When prompted which drug is more effective, people would typically interpret this table to be in support of drug A. Only between 30% and 40% of people correctly interpret covariation information presented in such a table, which is even lower than if they were guessing [4, 5]. This seems to be due to people assigning a high relative importance to the upper left cell’s absolute value rather than the more illuminating cross-row ratios [6]. Thus, a lobbyist working for the company that produces drug A could hypothetically (mis-)use this table to support the claim that their company’s drug was superior, even though the claim is not only not supported by the evidence, but even directly contradicted by it. In this study, we aim to answer the question of whether people are more careful in their evaluation of such statistical evidence when it is used to support a claim by a source who likely has a manipulative intent due to a personal bias.

**Table 1:** Number of sick people who took drug A or B and did or did not get better.

	Drug A	Drug B
Disease gets better	98	63
Disease does not get better	49	21

### Epistemic trustworthiness: The role of commercial interests

Source features, especially those that relate to the trustworthiness or credibility (both terms have been used interchangeably in the literature) of a source, have been identified as an important factor affecting reasoning about information of various kinds [7, 8]. Particularly in regards to science information, citizens often need to judge the trustworthiness of scientific sources instead of directly judging the credibility of scientific claims [9, 10]. Such trustworthiness judgments are made by reasoning about whether a source has the necessary expertise to provide correct information and whether the source is likely willing to do so; this second requirement is dependent on whether a source adheres to certain ethical standards and has the goodwill to provide true information (integrity and benevolence, respectively, in the following) [11].

Apart from the expertise of a source, which is the necessary condition to make trust possible, the motivation of a source is one of the most important factors people use to judge trustworthiness, especially in regards to financial interests. Large-scale representative surveys in Germany, Sweden and Australia found that scientific research was deemed less credible when it was either privately funded or conducted at companies [12–15]. Further, in a series of experiments where the professional affiliation of an expert source was changed between groups, scientists working as lobbyists were perceived as having less integrity, less benevolence and being more manipulative compared to publicly funded scientists [16–18]. Finally, people view scientists as being more strongly motivated by intrinsic motivations (which were mainly epistemic) compared to extrinsic motivations (such as money & power) but this difference is also present for scientists working at corporations [19]. Overall, people are able to identify conflicts of interests arising from financial influences on science, and rate sources, which are conflicted in this way as less trustworthy.

### Epistemic vigilance: Reasoning about an untrustworthy source’s claim

Perceptions of source trustworthiness in turn affect evaluations of claims or information stemming from that source [7, 20–23]. Most research has scrutinized the direct effect of source features on trustworthiness and credibility evaluations but there is evidence that these features can affect the quality of reasoning in experimental tasks, particularly when a source is perceived as not trustworthy.

For example, Copeland, Gunawan, and Bies-Hernandez [24] presented participants with two individuals’ inferences based on a fictitious survey of a small town. These inferences were syllogisms (e.g. *“Some married people are skiers*. *All skiers are tennis players*. *Some tennis players are married people*.*”*) and the participants had to indicate whether the drawn conclusion was correct. The authors manipulated the description of the source in a within-participant design. In experiment 1, participants were given such inferences from a trustworthy source (a highly regarded community member) and an untrustworthy source (a corrupt local politician). While there was no significant difference in accuracy for logically necessary syllogisms (which had a justified conclusion), participants performed better for syllogisms that were not justified when they were made by an untrustworthy source. That is, participants were more likely to correctly indicate that a drawn conclusion was wrong when the conclusion was made by the untrustworthy source. In experiment 2, Copeland et al. [24] manipulated the source information in a way where one source was an expert (a psychology professor experienced in working with survey data) and the other was a non-expert (a local mechanic who did not get into college). They observed an effect that was consistent with experiment 1: participants were more likely to indicate correctly that a drawn conclusion was wrong when the conclusion was made by the non-expert source.

In a replication and extension of this study, Boucher [25] did not find the source expertise effect for her entire sample but only for those participants who scored low on the cognitive reflection test (CRT). The CRT measures the general tendency to engage in effortful reasoning [26, 27]. Therefore, when participants who had a lower threshold to engage in effortful reasoning were excluded from the analysis, the remaining participants were those who applied effortful reasoning strategies selectively for the non-expert source.

These findings show that when people expect a non-trustworthy or non-expert source to be wrong, they scrutinize the claims made by that source more thoroughly and are thus able to identify logical inconsistencies. Since in these cases the source information was not enough to establish the epistemic validity of the source’s claim, or even indicated the claim to not be valid, people use other means (in this case more careful reasoning) to evaluate the validity of the claim. Relying on testimony by others can only be an epistemically valid strategy if there is a cognitive mechanism to identify untrustworthy sources and in turn become more attentive, or vigilant towards claims by such sources. Sperber et al. [28] call this epistemic vigilance which they describe *“not [as] the opposite of trust; [as] the opposite of blind trust”* (p. 235). They argue that trustful communication is only possible under the condition that humans possess certain cognitive mechanisms to identify what is untrue. Such a mechanism could be sensitive to source information and become active when it is likely that the source is misleading either due to lack of expertise, or on purpose. It is feasible that such a mechanism would also lead to scrutinizing the evidence underlying a claim, if possible. For epistemic vigilance to be directed at a risk of deception, people must possess an understanding of the source’s motivations (i.e. *does this claim serve the source’s interests*?), as well as of the source’s trustworthiness, particularly in regards to benevolence and integrity (i.e. *would the source deceive to further their interests*?). According to Sperber et al. [28], epistemic vigilance is not only sensitive to the risk of deception but when it does become active due to that risk, people would look to falsify the possibly deceptive claim with the available information. Further, since the function of epistemic vigilance is to limit the potential hazards of relying on testimony, when it is targeted at a possible risk of deception, it stands to reason that higher cognitive effort would be exerted to falsify but not to verify the possibly deceptive claim.

The concept of epistemic vigilance is a more general theoretical construct but its implications for how people deal with the risk of being misinformed is important, particularly in today’s digitalized society where competing claims are plentiful and source information is sparse or unreliable. Therefore, we aim to test these implications in regards to a situation where people are faced with a vested-interests source who (mis-)uses statistics to support a claim.

### The present study

In order to investigate such a situation, we conducted an online experiment in which we presented people with a source's claim and a contingency table. The claim is either in support or against a ban of an unhealthy product and refers to the contingency table as supporting evidence. However, the claim is not actually supported by the contingency table but even contradicted by it (as in the example surrounding Table 1). Therefore, when the claim was in favor of the ban of the product, the contingency table indicated that the product had some positive effects; and when the claim was against the ban, the contingency table indicated that the product had negative effects. We chose to include a task involving a contingency table in our study because such tasks have been widely used in research on statistical reasoning in the past [4, 5, 29] and because it involves overcoming certain typical response tendencies. This allows insights into possible changes of the reasoning process from one experimental condition to another and sets it apart from the rating-style items, which are more typically used in research on source effects.

We also manipulated the source to be more or less trustworthy due to vested interests. The source was introduced as either a university scientist, an industry lobbyist or a public health insurance speaker (HIS). A publicly funded scientists’ job is the production and dissemination of knowledge, which is why in a debate about the effects of an unhealthy product, we assume that people do not view him or her as having vested interests. Both an industry lobbyist, and a public health insurance speaker, though, have certain aims that go beyond purely epistemic aims and these could lead them to misuse statistics in order to support their position.

Here it is important to consider that trustworthiness evaluations are domain-specific. For instance, one might be vigilant towards the claim by an oil company lobbyist denying global warming, because the issue of global warming is related to her vested interests, but this vigilance might not extend to claims by the same person about quantum physics. Even further, one likely would not doubt her claim at all if she claimed global warming was indeed happening, thus taking a position counter to her supposed vested interests [30]. More generally, claim content and source characteristics interact both in regards to evaluations of the claim, as well as of the source [31–33].

For epistemic vigilance to be directed at a possibly deceptive claim, the source must be perceived as non-trustworthy and the claim as being in line with the source’s interests. As previously discussed, scientists are generally seen as trustworthy and their aims as mainly epistemic. Therefore, regardless of whether the scientist would argue for or against the unhealthy product, people would likely not become epistemically vigilant and thus not look to falsify the claim. Industry lobbyists on the other hand are seen as less trustworthy, and in the context of our experiment, the lobbyist would be motivated to misrepresent statistics to defend his company’s product. Further, since the product in question is unhealthy, a health insurance speaker could also have an interest to misrepresent statistics to make the product seem even worse and thus encourage a ban. However, while there is ample evidence that lobbyists are perceived as being less trustworthy than scientists, it is unclear whether a spokesperson for a public service such as health insurance is viewed in the same way. We included this source condition because a source’s vested interests are not always as immediately obvious as they are for industry lobbyists. Therefore, including this condition could help understand the conditions under which a possible effect of epistemic vigilance as a response to a vested-interest source’s claim occurs.

Importantly, while the hypothesized effect of epistemic vigilance is not dependent on the incongruence between the claim and the evidence (i.e. the suspicion that a source is deceptive does not have to be true), in our experimental paradigm, it is important that the contingency table at first glance (i.e. according to the typical, incorrect interpretation) seems to be in support of the source’s claim. If this were not the case, that is if at first glance the likely deceptive source were contradicted by the table, there would possibly be no need to further investigate the contingency table. Epistemic vigilance, if it is directed at the risk of deception would likely modulate reasoning in a direction of falsifying the claim [28]. Thus, the following hypotheses are formulated for the case where evidence contradicting a source’s claim is not available without careful reasoning. We would expect people to become vigilant towards the claim and more carefully reason about the contingency table when a vested-interest source argues in accordance with his or her interests but become less vigilant when a vested-interest source argues against his or her interests but we would expect no differences for the neutral source (*Hypothesis 1*; H1).

Being vigilant towards a source’s claim can only provide the motivation to more carefully reason about the contingency table. To solve a task such as the contingency table, people also need numerical ability. Therefore, the effect of becoming vigilant should be most pronounced for people with a high numerical ability. Such an effect has previously been found in the polarization of high-ability subjects in a motivated reasoning experiment [5]. We would therefore expect the effect of vigilance on reasoning to be most pronounced for individuals with higher reasoning ability (*Hypothesis 2*; H2).

Importantly, in regards to H1 and H2, we expect people to become vigilant and reason more carefully about the contingency table when a source behaves *as expected*. There is also evidence that social reasoning is affected when the reasoning target behaves unexpectedly. For instance, person memory is increased for people who behave in a trait- or stereotype-inconsistent way [34]. However, we don’t know of studies showing that this effect on recall is also present for claims from unexpectedly behaving sources. Even if people would become more attentive in such a case to resolve the discrepancy between what was expected and what was observed, the contingency table in our experiment might not be perceived as a relevant piece of evidence to this process. If people did find out that the source misrepresented the data in the table but in order to support a claim which went counter to suspected motivations, this would only make the source behavior even more unexpected. Especially considering that past research [24] has found that when sources behave in an untrustworthy manner, people are better at logically reasoning about the premises of claims for that sources, we find that there is stronger evidence for our expected effect: that people would be better at mathematically reasoning about the values in a contingency table when a combination of source features and source behavior indicate that they might be used to mislead recipients.

Finally, there could also be an effect on participants’ direct evaluations of the source when they find out that a source misrepresented statistical evidence to support a claim. We do not have specific hypotheses but are interested in whether people would view this as a deliberate attempt at misinformation (*Research Question 1*; RQ1) and secondly, whether trustworthiness is affected (*Research Question 2*; RQ2).

## Methods

The study was approved by the ethics commission of the University of Münster’s Institute of Psychology (identifier: 2019-16-LG). Participants had to give consent three times throughout the experiment. On the very first page after a short introduction into the procedure, they had to indicate that they were willing to take part in the experiment. After the last dependent variables were filled out, they were debriefed and were prompted to indicate whether their responses could be used for scientific analyses and whether they understood that all materials shown to them throughout the experiment were fictitious.

### Design

The experiment used a 3x2 between-participant experimental design with source affiliation (lobbyist, HIS, and scientist) and direction of claim (pro chocolate milk, contra chocolate milk) as independent variables.

### Materials

All materials were presented in German.

#### Stimuli and intervention

**Fig 1:**
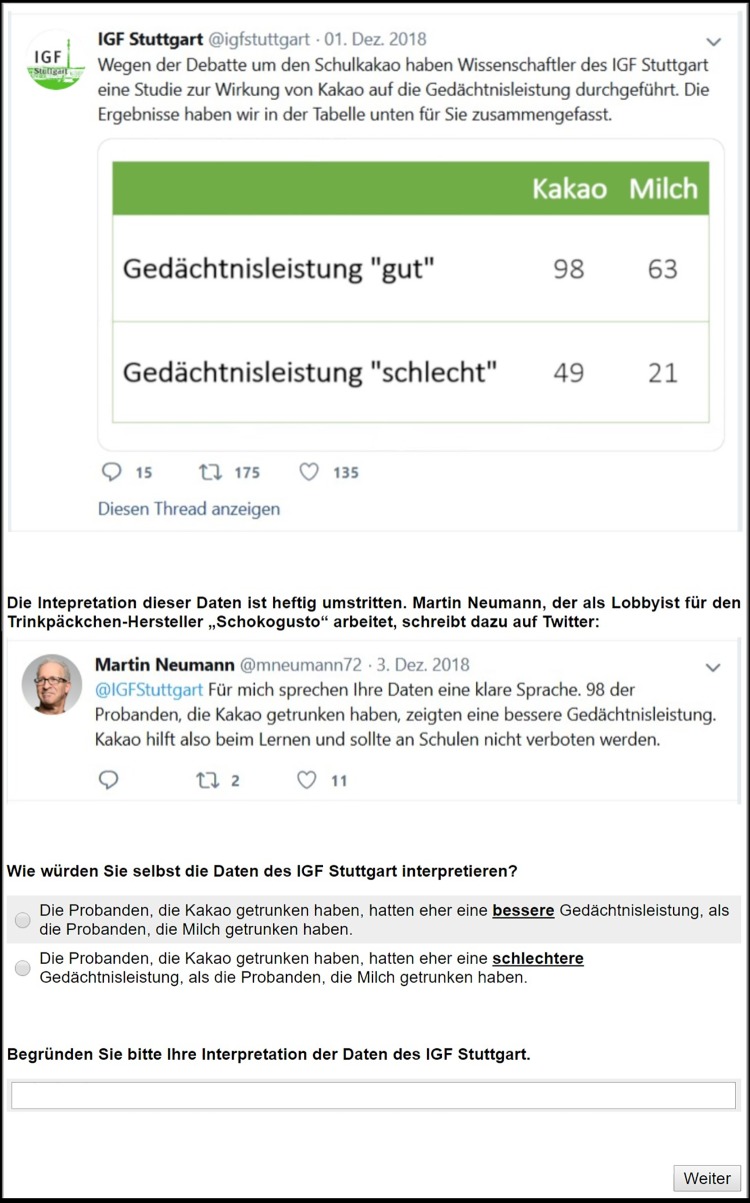
Screenshot of experimental setting.

Since there is ample evidence that sugar-sweetened beverages have negative health effects, we chose chocolate milk to represent the unhealthy product [35]. The main intervention included a short introductory text about a controversy surrounding a ban on chocolate milk in school cantinas and two screenshots of fictitious tweets. The first screenshot was from the fictitious Institute of Health Research Stuttgart (IHR Stuttgart) and included results of a study about the effects of chocolate milk on memory capacity. This tweet included mainly a contingency table detailing the results (see Fig 1). The second tweet was by a person called Martin Neumann who commented on the results of the study. Martin Neumann’s tweet and twitter handle were created for this study.

Depending on the source affiliation condition, this person was introduced as either a university scientist (neutral source), a public health insurance spokesperson or a lobbyist for a fictitious chocolate milk producer (vested-interest sources). Depending on the claim direction condition, the person interprets the results as showing either that chocolate milk improves memory capacity (pro-chocolate milk) in comparison with pure milk or vice versa (contra-chocolate milk). However, regardless of condition, the source’s interpretation of the contingency table is incorrect.

#### Contingency table task

The contingency table task is a classic heuristic-and-biases task and has been previously used to study the difference between heuristic and analytic reasoning [5, 27, 29]. Past research suggests that people are systematically incorrect on this task with past studies finding a baseline of between 31.7% and 41% [4, 5] correct responses in non-intervention groups. The contingency table in our task was also constructed in such a way that the highest absolute value indicated that chocolate milk improved memory performance compared to regular milk (or vice-versa, depending on condition).

Our main dependent variable was whether participants accurately judged the contingency table. To this end we asked them: “*How would you yourself interpret the IHR Stuttgart’s data*?” with the response options being, “*The participants who drank chocolate milk had* ***better*** *memory performance compared to the participants who drank milk*”, and, “*The participants who drank chocolate milk had* ***worse*** *memory performance compared to the participants who drank milk*”. In conditions where the contingency table was flipped so that milk was the incorrect response the response options of this item were formulated such that milk was mentioned first, i.e. “*The participants who drank milk had* ***better*** *memory performance compared to the participants who drank chocolate milk*”. Such a forced-choice design was used in previous implementations of the contingency table task [5]. The data presented in the contingency table were fabricated and do not reflect real scientific findings on the topic.

#### Trustworthiness

We assessed trustworthiness with the Muenster Epistemic Trustworthiness Inventory [11]. It consists of three scales: expertise with six semantic differentials (e.g. *incompetent*-*competent*), integrity with four semantic differentials (e.g. *unjust*-*just*) and benevolence with four semantic differentials (e.g. *unethical*-*ethical*). All responses were recorded on a 7-point scale. The internal consistencies of all three scales were good to excellent (*α*_*expertise*_ = .93; *α*_*integrity*_ = .88; *α*_*benevolence*_ = .87).

#### Numeracy

Performance in the contingency table task is correlated with numeracy and cognitive reflection as measured by the cognitive reflection test [5, 26]. In our study, we included four items from a general numeracy scale [36]. Since the validity of the CRT suffers when participants already know the items [37] we included two items of the more recently developed long version of the CRT [38]. Numeracy was assessed as the total number of correctly answered items and ranged therefore from 0 to 6.

#### Perceived deception attempt

Since the source’s claim in the experiment was incorrect, we also wanted to know whether participants perceived this as an attempt at deception (or intentional misinformation). Therefore, we asked them to indicate their agreement with *“Martin Neumann wanted to intentionally deceive the readers of his twitter message”* on a 5-point scale.

#### Memory check

We asked participants to indicate the profession of the source. Response options included the three source affiliations used in the experiment (lobbyist, scientist and HIS) and one distractor item (school principal). Such source memory tests are often used in research on source effects [39].

#### Further measures

We also asked participants to justify their response to the contingency table task in an open text box. Further, we measured propensity to trust with 6 items and attitude towards a chocolate milk ban with 2 items, responses to which were recorded on a 5-point scale. None of these further measures were analyzed here, as they would have surpassed the scope of the study.

### Procedure

Participants completed the survey online. The recruiting call included instructions to complete the survey in a quiet environment and on a PC, laptop or tablet. After filling out the informed consent form, the stimulus introduction and screenshots, including the contingency table task, were presented to the participants. This was presented on the same page so participants could refer back to the contingency table when thinking about their answer. Then, participants rated source trustworthiness, topic attitude and filled out a memory check. After that, participants completed the numeracy and propensity to trust questionnaires and, lastly, demographics. We chose to present the numeracy and propensity to trust scales after the intervention even though they were covariates. This was for two reasons: we wanted to avoid tiring out participants from the start, especially since the numeracy items were found to be cognitively challenging in pre-tests, and secondly, because both scales could activate unwanted cognitive schemas; the numeracy scale could have made participants focus on the mathematical side of the contingency table task by default and the propensity to trust scale could have made participants privy to the notion that the experiment was about trust, thus inducing unwanted experimenter demand.

### Participants

We recruited a total of *N* = 507 participants through testingtime.com. According to Testingtime’s own information, they have a pool of about 350,000 participants, mainly from German-speaking countries, 35% of which are between 30 and 50 years of age and 41% of which hold an academic degree. The sample consisted of 271 females (53.45%) and had a mean age of *M* = 32.97 (*SD* = 10.79). 26.63% of participants were students. On average, participants took 10 minutes and 27 seconds to complete the study.

An a-priori power analyses was conducted with G-power 3.1 [40]. We did not know how large the effect of epistemic vigilance on the solving rate of the contingency table would be. Another study using the same dependent variable reported a baseline performance of 31% correct solvers, and a logit coefficient for the effect of identity-protective cognition of 1.28 (*OR* = 3.60) [5]. For this effect size (with *α* = 5%, *β* = 5%, *R*^2^ other X = 20%, X-distribution = binomial and *π* = 0.5) the recommended total sample size is *N* = 141. As we suspected the source effect in our study to be smaller, we ran the analyses again with *OR* = 2. This yielded a recommended total *N* = 483.

We only wanted to include participants in the analysis who correctly remembered the source affiliation in their condition (see below for further information on the memory check item). However, only 57.79% of participants correctly answered the memory check. This led to a sample size of *N* = 293 (150 female). Since such an amount of participants had to be excluded, it is crucial to know whether the remaining participants differ from those that were left out in some meaningful way. For this, we compared the participants who answered the memory check correctly with those who did not in regards to age, gender, education, numeracy and regarding the solving rate of the contingency table task. The only statistically significant difference was in regards to age with excluded participants being slightly older (*ΔM* = 2.52, 95% CI [0.58, 4.45], *t*(420.69) = 2.55, *p* = .011). Overall, with the measures available to us, we do not find meaningful differences between the remaining and excluded participants beyond source memory. Since our experimental effect hinges on participants paying attention to the source, we exclude the participants who did not answer the memory check item correctly.

However, with such an amount of excluded participants, it is also important to know whether the likelihood of correctly remembering the source depended on the experimental manipulation, particularly on the source conditions. Therefore, we conducted a logistic regression analysis predicting the likelihood of correctly remembering the source with source condition and claim direction as independent variables (see S1 Table) for full model results). The results show that people were less likely to remember the source when it was a HIS compared to when it was a scientist or lobbyist.

### Data analysis

We initially planned on running successive logistic regression analyses with source condition, claim direction and numeracy as independent variables, as well as their interaction terms which we had also based our power analysis on. However, since the excluded participants were non-randomly distributed among the source conditions, we do not report tests comparing one source condition to another. To test whether performance in the contingency table task significantly depended on our experimental manipulation, we instead performed separate Fisher tests for each source condition. To test the moderating effect of numeracy, we computed logistic regression analyses. To test whether correctly solving the contingency table task affected the perception of a deception attempt or the source trustworthiness, we conducted within-condition t-tests comparing solvers and non-solvers. Categorical variables were dummy-coded where needed. All analyses were performed using R [41].

## Results

### Contingency table task performance

**Fig 2:**
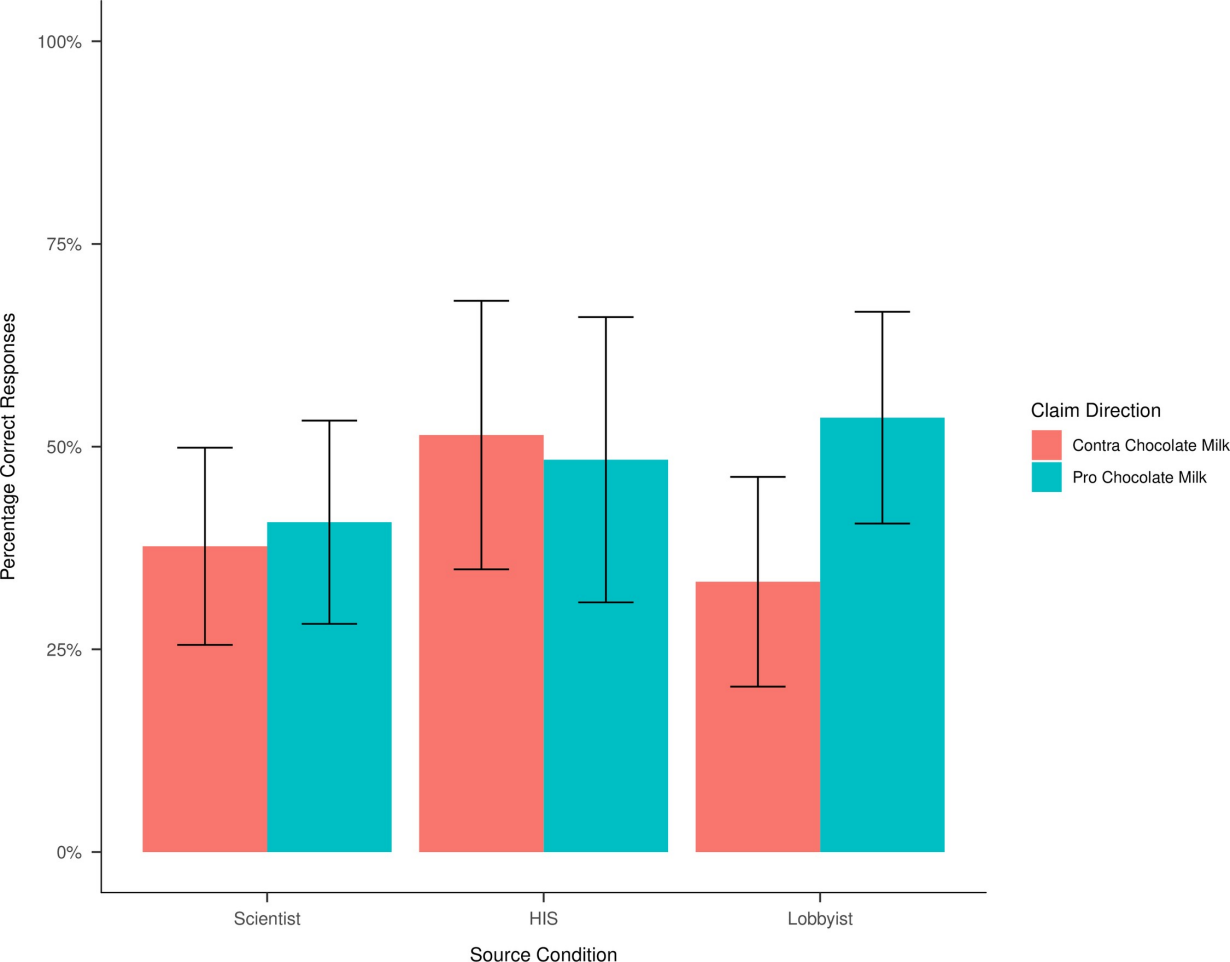
Contingency table task performance. HIS = Health Insurance Speaker; Confidence intervals were computed on the 95% confidence level.

Overall, 127 (43.34%) of participants answered the contingency table task correctly. Fig 2 shows the percentage of correct responses per condition. We expected better performance when the HIS argues against chocolate milk (contra) compared to when he argues for chocolate milk (pro), and when the chocolate milk lobbyist argues for chocolate milk compared to when he argues against chocolate milk but no difference between claim directions for the scientist (*H1*).

To test H1, we computed Fisher’s exact tests of the 2x2 contingency tables containing the frequency of solvers and non-solvers depending on claim direction for each source separately (one-sided for the vested-interest source groups, two-sided for the neutral group). The results show that claim direction significantly affected solving only for the lobbyist source condition (*n =* 107, *OR*_*Pro/Contra*_ = 2.31, *p* = 0.03) but not for the HIS condition (*n =* 66, *OR*_*Pro/Contra*_ = 0.89, *p* = 0.50) nor for the scientist group (*n =* 120, *OR*_*Pro/Contra*_ = 1.13, *p* = 0.85). Remarkably, participants’ odds for correctly interpreting the contingency table were more than doubled, when the lobbyist argued for chocolate milk compared to when he argued against it. However, since performance did not depend on claim direction for the HIS condition, H1 was only partially confirmed.

Further, we expected that the effect of claim direction would be stronger among participants with higher numeracy scores (*H2*). We tested this assumption by comparing two logistic regression models predicting correct responses, but only in the lobbyist condition since we found no effect of claim direction in the HIS condition. The first model contained numeracy and claim direction as predictors. The second model additionally included the two-way interaction between numeracy and claim direction (see Table 2 for model details).

**Table 2:** Logistic regression predicting contingency table task performance in the lobbyist condition (N = 107).

	Model 1				Model 2				Model 3			
	*β*	*SE*_*β*_	*z*	*p(z)*	*β*	*SE*_*β*_	*z*	*p(z)*	*β*	*SE*_*β*_	*z*	*p(z)*
Intercept	-0.69	0.30	-2.33	0.020	-2.81	0.70	-4.03	<0.001	-1.48	0.68	-2.19	0.028
Pro	0.84	0.40	2.09	0.037	0.66	0.44	1.52	0.129	-3.68	1.62	-2.27	0.023
Numeracy					0.53	0.14	3.77	<0.001	0.21	0.15	1.36	0.173
Pro x Num.									0.99	0.35	2.81	0.005
χ^2^_inc._	4.48 (p = 0.034)				18.47 (p < 0.001)				9.88 (p = 0.002)			
AIC	146.27				129.80				121.92			
BIC	151.62				137.82				132.61			

*Pro = Pro Chocolate Milk claim; Num*. *= Numeracy*

**Fig 3:**
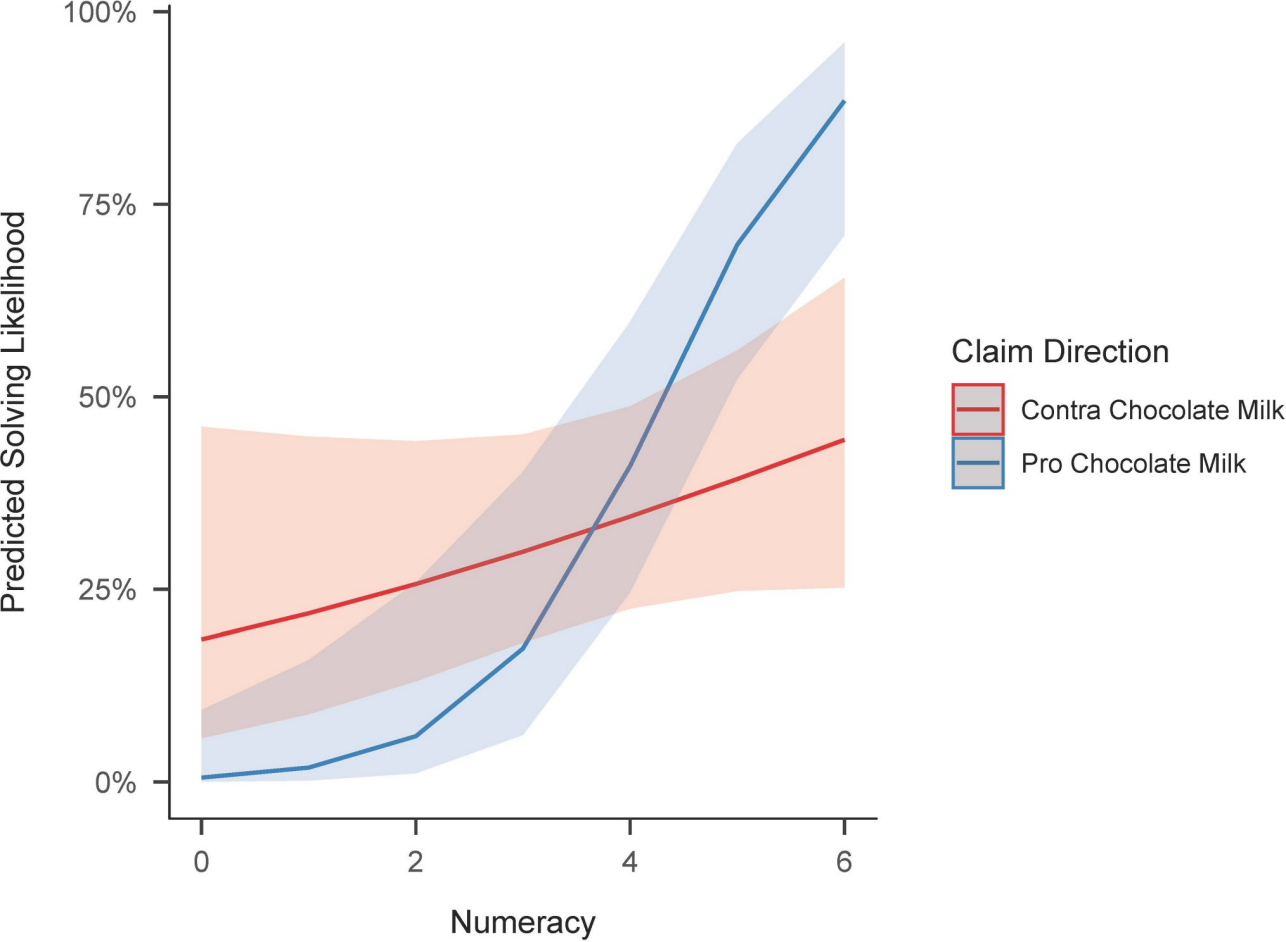
Predicted contingency table task performance in the lobbyist source condition. HIS = Health Insurance Speaker; Shaded areas around graphs represent the standard errors of the marginal effects.

The coefficient estimate of the interaction term indicates that numeracy improved performance more strongly when a lobbyist argued in favor of chocolate milk. Since this value does not aptly capture the non-linear property of the link function, we plotted the predicted probabilities of successfully completing the contingency table task for different numeracy scores in the different experimental conditions in Fig 3. Importantly, the confidence bands of the graphs are quite large and overlap in several places. Nevertheless, the significant coefficient and model test lead us to say that H2 was also partially confirmed.

### Further analyses: Effects of solving the contingency table

Since we would not have had at least 30 participants in each condition if we were to include correctly solving the contingency table task as another factor for variation analysis, we computed t-tests comparing those who correctly solved the contingency table task (thus finding out that the source’s claim was incorrect; called “solvers” in the following) with those who did not (see Table 3). Since we computed six t-tests per dependent variable, we used a Bonferroni-corrected *α* = 0.8% to avoid first-type error accumulation.

**Table 3:** Mean differences between solvers and non-solvers.

		Perceived Deception			Expertise			Integrity			Benevolence		
Condition		*ΔM*	*t*	*p(t)*	*ΔM*	*t*	*p(t)*	*ΔM*	*t*	*p(t)*	*ΔM*	*t*	*p(t)*
**Scientist**	Pro	-0.13	-0.43	.666	**1.73**	5.04	< .001	**1.20**	3.76	< .001	**0.94**	3.27	.002
	Contra	-0.70	-2.52	.015	**1.03**	2.82	.007	**1.03**	3.58	.001	0.63	2.20	.034
**HIS**	Pro	0.00	0.01	.992	1.39	2.76	.011	1.05	2.46	.020	0.30	0.73	.469
	Contra	-0.19	-0.53	.600	**1.13**	2.88	.007	0.54	1.40	.171	0.21	0.57	.573
**Lobbyist**	Pro	**-1.00**	-3.71	< .001	0.83	2.56	.013	**1.22**	4.10	< .001	**0.87**	3.08	.003
	Contra	0.00	0.00	1.000	**1.26**	4.87	< .001	**1.21**	3.51	.001	**0.92**	2.87	.007

Positive values indicate that Non-Solvers had a higher mean compared to the Solvers; negative values indicate the opposite. Mean differences which were statistically significant are printed in bold type. HIS = Health Insurance Speaker

#### Perceived deception attempt

Solvers perceived an attempted deception by the source only when the source was a lobbyist arguing in favor of chocolate milk.

#### Trustworthiness

Compared to non-solvers, solvers generally rated the source as being less competent, having less integrity and being less benevolent in all conditions. For expertise, the difference was not statistically significant for the HIS arguing for chocolate milk and lobbyist arguing for chocolate milk. For integrity, the difference was not statistically significant for the two HIS conditions. For benevolence, the difference was not statistically significant for the two HIS conditions, nor for the scientist arguing against chocolate milk.

## Discussion

This study dealt with the question whether people examine statistical evidence used to support a claim more carefully when the claim’s source likely has a manipulative intent due to a personal bias. We presented people with a claim about an unhealthy product that referred to a contingency table as evidence and was made by a source that either had vested interests in the matter (an industry lobbyist and a public health insurance speaker) or was neutral (a university scientist). We hypothesized that when a source with vested interests made a claim that was in line with these interests, people would examine the supposedly claim-supporting evidence in the contingency table more critically compared to when the same source made a claim that was counter to these interests. For the neutral source, the claim direction should not matter (*H1*).

Indeed, for a lobbyist source, participants were more likely to solve a contingency table task when he argued for his companies’ product compared to when he argued against it, while for the neutral source, a university scientist, the claim direction did not matter. For the health insurance speaker, whom we suspected would be perceived as biased against the lobbyists’ unhealthy product, correctly interpreting the contingency table did not depend on the claim direction either. We also expected the effect of claim direction to be dependent on numeracy (*H2*). Indeed, high-ability individuals were more likely to successfully employ analytic reasoning strategies when the lobbyist claimed that his companies’ product was superior compared to when it was not.

These findings offer some support for the presence of a vigilance mechanism that leads to more sophisticated reasoning about a biased source’s claim. This mechanism could be explained by people becoming more motivated to be accurate in their reasoning [42], especially when one considers the moderating effect of numeracy. Since high-numeracy individuals have the necessary ability to correctly interpret the contingency table, whether they do or do not depends mostly on their motivation. Potentially, this motivation was provided by the vigilance towards the lobbyist’s biased claim. This can also be seen as complementary to the finding by Boucher [25] that the source credibility effect was only present for individuals *low* in reflective thinking; they were not necessarily less *able* but less *motivated* reasoners. Similarly, Pennycook and Rand [43] identified that people high in reflective thinking are less likely to believe “fake news” stories, regardless of whether these stories align with their political leanings. Again, a reflective or critical reasoning style is more of a motivational disposition but both motivation and general cognitive ability are needed to properly engage with second-hand information. Indeed, Ståhl and van Prooijen [44] found that a motivation to engage in critical thinking together with a lack of cognitive ability can even lead people to develop a conspiracist mentality.

Our further analyses dealt with the question how interpreting the contingency table differently than the source (which, again, was the normatively correct interpretation of the table) would affect the perception of the source as deceitful (*RQ1*) and more or less trustworthy (*RQ2*). Participants who correctly interpreted the table viewed the source’s claim as a deliberate attempt at deception only when the source was a lobbyist arguing for his company’s product. Regarding the effect on trustworthiness, ratings of expertise, integrity and benevolence seemed to generally suffer when people interpreted the table differently than the source. Interestingly, however, the lobbyist’s perceived expertise was affected much less when he argued for his company’s product compared to when he argued against it. This would make sense if people assumed that the lobbyist had misrepresented the statistical evidence on purpose because his “error” wouldn’t have been a matter of expertise then. Both of these findings support the notion that participants viewed the lobbyist as someone who would intentionally misrepresent statistical data to further his interests which would lead them to be vigilant towards the lobbyist’s claim if the claim was in line with his interests. Potentially, there were no similar expectations of the participants regarding the health insurance speaker; either he was not perceived as having vested interests or at least none strong enough to misrepresent statistics for. This could be the case because a potential ban of chocolate milk in schools is just one of many issues that are relevant for public health. In comparison, for a manufacturer of chocolate milk cartons such a ban would have a strong, direct and negative effect on business. However, this is pure speculation; due to our experimental design, we could not have asked participants about the suspected motivations of the sources prior to giving their response in the contingency table task.

More generally, these findings also confirm that source credibility is negatively affected when sources make incorrect claims [33, 45]. Our findings confirm this for the case where the claims were found out to be incorrect by the participants via their own reasoning, and further add to the understanding of trustworthiness judgments being subject to a feedback mechanism that continually updates them based on behavior of the source [46]. Interestingly, the solvers did not get a feedback that their interpretation of the contingency table was correct. Further, it is unclear whether the non-solvers also updated their trustworthiness judgments based on the “feedback” that the source came to the same conclusion as they did when reasoning about the contingency table.

One important, if incidental finding for the practical contextualization of our results is that only 57.79% of participants noticed or remembered the source manipulation. There is evidence that people generally do not attend to source features except when prompted, or when there is a conflict present [47, 48]. In this study, we did not prompt people to reason about the source of the claim, rather we prompted a response in regards to the contingency table. This added demand could have distracted some people from the information about the source. Further, people are better at remembering information about sources which have shown dishonest behaviors in the past [39]. Interestingly, in our experiment, source memory was the same for lobbyists (which are seen as less trustworthy in general) and scientists but not for the health insurance speaker. Taken together, it is still an interesting finding for the research on sourcing behavior that the capacity to become vigilant towards source’s claims based on the interplay between source characteristics and source behavior can be an outcome of sourcing behaviors. However, it is therefore also important to consider whether enough people even pay attention to sources for this effect to have a meaningful for the reception of information from sources in online contexts.

### Limitations

We did not foresee that more than 40% of participants would not remember the critical source manipulation. Exclusion of these participants diminishes both the statistical power of our tests, as well as the generalizability of the results since we can only make statements about those people who were attentive enough to remember the professional affiliation of the source. Arguably, in real-world contexts the professional background of a source can at times be even more difficult to ascertain. Further, people might reason about real tweets differently than about the ones we have constructed for this study. However, they were indistinguishable from real tweets and thus might have made the otherwise text based information a little bit more believable for participants.

Another limitation is that we did not control for the prior attitudes of our participants in regards to the debate surrounding chocolate milk. This could have affected the evaluation of the source’s claim. However, there are some reasons why systematic attitude effects might not be so severe in our study: firstly, the topic of the cover story wasn’t directly about the health effects of chocolate milk but about potential memory benefits which were fabricated. Secondly, the debate about the health effects of chocolate milk and a potential ban in school cafeterias were not as widely discussed in the press as more controversial topics (e.g. vaccinations). Finally, we had a completely random assignment of participants to experimental conditions.

There is also a limitation regarding the design of the main dependent variable. We used a forced-choice design in the response to the contingency table where participants had to decide whether the table supported the notion that chocolate milk was *superior* or *worse* than milk. However, some people might have (mis-)understood the table as supporting neither direction. While it is possible that this group was evenly split among both response options, it could also be that they would then systematically choose one option over the other. If this in turn was dependent on the experimental condition they were in, then that could have skewed our results.

### Conclusions, future research directions & implications

In a society characterized by a large degree of division of cognitive labor, people need to engage with expert sources to make decisions based on knowledge that is difficult to understand [49]. One challenging aspect is to identify biased sources and to appropriately deal with claims made by those sources. In this experiment, both the appropriate activation of vigilance in response to a biased source’s claim, as well as more general cognitive abilities led to more sophisticated reasoning about scientific evidence in an online scenario.

The interpretation of the pattern of results as being due to the proposed epistemic vigilance mechanism is still unclear in some regards. For instance, we did not include an experimental condition in which vigilance towards the source was forced, for instance via the instructions. Including such an explicit instruction in future research could be an illuminating next step for such an experimental paradigm, particularly considering that many participants did not remember the source affiliation in our experiment. More generally, epistemic vigilance should affect reasoning about all kinds of claims, even those that turn out to not be false. The understanding of the effect of epistemic vigilance on reasoning would therefore benefit from future implementations of similar experimental designs with different dependent variables. While further research is needed to fully understand the epistemic vigilance mechanism, there are still some preliminary implications, which can be drawn from our results.

Improving general abilities can allow for more effective scrutinizing of claims made by biased sources. While it is important to note that general cognitive abilities will not always lead to more enlightened processing of scientific information, as it can even increase political polarization [5], it might also provide the necessary background to appropriately deal with biased sources. However, not all people spontaneously attend to source features. Therefore, measures to improve sourcing behaviors [47] would be beneficial for the public’s engagement with science information not just to more appropriately judge what is right, but also to identify situations in which vigilance towards a vested-interest source’s claim is warranted.

Even further, such abilities could help not just in reasoning about science information in general, but also specifically in regards to dealing with science denialism. Misinformation by global warming deniers attacking a message about the scientific consensus on global warming was found to be less effective if the message was preceded by the statement *“some politically motivated groups use misleading tactics to try to convince the public that there is a lot of disagreement among scientists”* [50]. It could be that when such vested interests are made salient, people become vigilant, as they likely did in our study, which can then lead to more sophisticated reasoning. This is indirect evidence that such “inoculation” attempts [50] could be successful in making people scrutinize claims made by likely biased sources.

In summary, when biased sources misrepresent statistics to support a biased claim, people are able to scrutinize the claim in front of them. However, this only holds when they are both attentive to source information in the first place, and have the necessary cognitive tools to do so.

## Supporting information

S1 Table Logistic regression predicting memory check performance (N = 507). Notes. HIS = Health insurance speaker; Pro = Pro Chocolate Milk; Scientist is the reference category for the source variable, Contra Chocolate Milk for the claim direction variable.
(DOCX)
