# Supplementary material for: Beware of vested interests: Epistemic vigilance improves reasoning about scientific evidence (for some people)
Source: PLoS One. 2020 Apr 15;15(4):e0231387. doi: 10.1371/journal.pone.0231387 (PMC7159212; doi:10.1371/journal.pone.0231387)
Supplement: S1 Table — Notes. HIS = Health insurance speaker; Pro = Pro Chocolate Milk; Scientist is the reference category for the source variable, Contra Chocolate Milk for the claim direction variable. (DOCX) [file pone.0231387.s001.docx]

# S1. Memory Check Analysis

We computed two logistic regression models predicting memory check performance, one with both source and claim direction as predictors and one with source, claim direction and the interaction term between source and claim direction as predictors. Categorical variables were dummy coded where necessary.

| Logistic Regression Predicting Memory Check Performance (N = 507) | | | | | | | | |
| --- | --- | --- | --- | --- | --- | --- | --- | --- |
|  | Model 1 | | | | Model 2 | | | |
|  | *β* | *SE_β_* | *z* | *p(z)* | *β* | *SE_β_* | *z* | *p(z)* |
| Intercept | 0.852 | 0.191 | 4.46 | <0.001 | 0.933 | 0.241 | 3.87 | <0.001 |
| HIS | -1.25 | 0.228 | -5.49 | <0.001 | -1.25 | 0.328 | -3.81 | <0.001 |
| Lobbyist | -0.223 | 0.231 | -0.962 | 0.336 | -0.467 | 0.330 | -1.41 | 0.157 |
| Pro | -0.0673 | 0.187 | -0.361 | 0.718 | -0.223 | 0.331 | -0.673 | 0.501 |
| HIS X Pro |  |  |  |  | -0.017 | 0.458 | -0.036 | 0.971 |
| Lobby X Pro |  |  |  |  | 0.486 | 0.464 | 1.05 | 0.295 |
| χ2inc. | 36.263 (p < 0.001) | | | | 1.553 (p = 0.46) | | | |
| AIC | 662.228 | | | | 664.675 | | | |
| BIC | 679.142 | | | | 690.046 | | | |
| Notes. *HIS = Health insurance speaker; Pro = Pro Chocolate Milk; Scientist is the reference category for the source variable, Contra Chocolate Milk for the claim direction variable.* | | | | | | | | |
